# Characterising an aromatase inhibitor resistant breast cancer cell line

**DOI:** 10.1186/1753-6561-6-S4-P3

**Published:** 2012-07-09

**Authors:** E Harris, D Varešlija, J O'Hara, A Hill, L Young

**Affiliations:** 1 Royal College of Surgeons in Ireland; 2 Endocrine Oncology Research, Department of Surgery, Royal College of Surgeons in Ireland

## Introduction

Aromatase inhibitors (AI) are a novel adjuvant endocrine treatment for estrogen receptor (ER)-positive, postmenopausal breast cancer. They function by inhibiting the aromatase enzyme that converts androgens into estrogens. AIs have demonstrated excellent efficacy in clinical trials and have shown supremacy over Tamoxifen. However, prolonged use of AIs can lead to acquired resistance. This resistance is characterised by aberrant ER signalling and crosstalk with growth factor pathways. CyclinD1 is currently being investigated in the lab in an AI resistant breast cancer cell line (Let-R) and there is evidence of differential gene expression when compared to classical genes such as pS2. In Let-R cells, cyclinD1 expression appears to remain estrogen regulated. It is thought that this is not solely regulated through ER but through estrogen signalling to c-jun N-terminal kinase (JNK). This study aims to characterise the Let-R cell line created in the lab and to optimise an ERα knockdown in Let-R cells.

## Methods

Parental MCF-7, aromatase-overexpressing (Aro) and Let-R cell lines were used to investigate protein expression levels of ERα, cyclinD1, JNK and c-jun using Western blots. Cells were transfected with ERα SiRNA to optimise the knockdown in Let-R cells.

## Results

Let-R cells have higher levels of ERα compared to Aros. CyclinD1 basal levels are elevated in Aro cells compared to Let-Rs. Levels of JNK and c-jun are higher in Let-R cells compared to Aros. A highly effective ERα knockdown in Let-R cells was optimized.

## Conclusions

The significant ERα knockdown achieved in the Let-R cells will enable further examination of knockdown effects on protein levels and mRNA expression. Elevated expression of ERα in the Let-R cells observed is consistent with the association between AI resistance and ER hypersensitivity. In resistant cells, the decrease in basal levels of CyclinD1 may be partly responsible for the lack of regulation of classical genes like pS2, previously detected by the lab. The elevated levels of JNK and c-jun observed in Let-R cells supports the theory of crosstalk between the ER and growth factor pathways in resistance. Furthermore, it would be of interest to accompany AI treatments with growth factor inhibitors to further our understanding of endocrine resistance.

